# AQP2 Plasma Membrane Diffusion Is Altered by the Degree of AQP2-S256 Phosphorylation

**DOI:** 10.3390/ijms17111804

**Published:** 2016-10-28

**Authors:** Eva C. Arnspang, Frédéric H. Login, Jennifer S. Koffman, Prabuddha Sengupta, Lene N. Nejsum

**Affiliations:** 1 Department of Clinical Medicine, Aarhus University, DK-8000 Aarhus C, Denmark; arnspang@kbm.sdu.dk (E.C.A.); frederic.login@clin.au.dk (F.H.L.); 2 The Eunice Kennedy Shriver National Institute of Child Health and Human Development, National Institutes of Health, Bethesda, MD 20892, USA; prabuddha.sengupta@gmail.com; 3 Department of Interdisciplinary Nanoscience Center, Aarhus University, DK-8000 Aarhus C, Denmark; jenniferkoffman@gmail.com

**Keywords:** diffusion coefficients, kICS: k-space Image Correlation Spectroscopy, MDCK, AQP2, kidney, urine, phosphorylation

## Abstract

Fine tuning of urine concentration occurs in the renal collecting duct in response to circulating levels of arginine vasopressin (AVP). AVP stimulates intracellular cAMP production, which mediates exocytosis of sub-apical vesicles containing the water channel aquaporin-2 (AQP2). Protein Kinase A (PKA) phosphorylates AQP2 on serine-256 (S256), which triggers plasma membrane accumulation of AQP2. This mediates insertion of AQP2 into the apical plasma membrane, increasing water permeability of the collecting duct. AQP2 is a homo-tetramer. When S256 on all four monomers is changed to the phosphomimic aspartic acid (S256D), AQP2-S256D localizes to the plasma membrane and internalization is decreased. In contrast, when S256 is mutated to alanine (S256A) to mimic non-phosphorylated AQP2, AQP2-S256A localizes to intracellular vesicles as well as the plasma membrane, with increased internalization from the plasma membrane. S256 phosphorylation is not necessary for exocytosis and dephosphorylation is not necessary for endocytosis, however, the degree of S256 phosphorylation is hypothesized to regulate the kinetics of AQP2 endocytosis and thus, retention time in the plasma membrane. Using k-space Image Correlation Spectroscopy (kICS), we determined how the number of phosphorylated to non-phosphorylated S256 monomers in the AQP2 tetramer affects diffusion speed of AQP2 in the plasma membrane. When all four monomers mimicked constitutive phosphorylation (AQP2-S256D), diffusion was faster than when all four were non-phosphorylated (AQP2-S256A). AQP2-WT diffused at a speed similar to that of AQP2-S256D. When an average of two or three monomers in the tetramer were constitutively phosphorylated, the average diffusion coefficients were not significantly different to that of AQP2-S256D. However, when only one monomer was phosphorylated, diffusion was slower and similar to AQP2-S256A. Thus, AQP2 with two to four phosphorylated monomers has faster plasma membrane kinetics, than the tetramer which contains just one or no phosphorylated monomers. This difference in diffusion rate may reflect behavior of AQP2 tetramers destined for either plasma membrane retention or endocytosis.

## 1. Introduction

Short-term urine concentration is regulated in the renal collecting duct principal cells in response to circulating levels of the antidiuretic hormone arginine vasopressin (AVP), which mediates water uptake from the renal ultra-filtrate. Water crosses the epithelial plasma membranes through aquaporin (AQP) water channels, driven by an osmotic gradient. AQPs are homotetrameric transmembrane proteins, expressed in multiple epithelial tissues including lung [1], sweat glands [2], skin [3], and kidney (for review [4]). Each monomer contains a pore selective for water (for review [4]).

**Figure 1:**
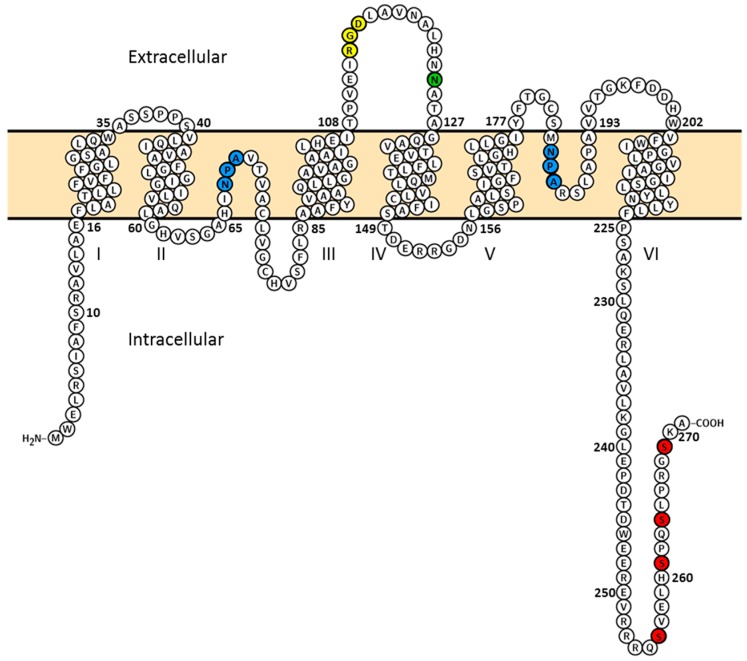
Membrane topology of rat aquaporin-2 (AQP2). Topology of rat AQP2 (Uniprot entry P34080) visualized with Protter [13]. AQP2 is an integral membrane protein with six transmembrane helices (I to VI). Two NPA motifs (blue residues) are found between the transmembrane helices II to III and V to VI. These NPA motifs are within two half-helices that bend into the plasma membrane to form a pore selective for water. It has been shown that the asparagine 124 (green residue) is *N*-glycosylated [14]. The RGD motif (yellow residues) in the extracellular loop between the transmembrane helices III and IV promotes interaction with integrin β1 [15,16]. Both the N and the C-termini are localized in the cytosol. AQP2 is phosphorylated at serine 256, 261, 264, and 269 localized within the C-terminal tail (red residues) [17,18,19,20,21,22].

Water enters the collecting duct principal cells via apical AQP2 [5] and exits via basolateral AQP3 [6] and AQP4 [7,8]. AQP2 localizes to subapical vesicles [5], the apical membrane [5], and—in some collecting duct segments—also to the basolateral membrane [9]. In the absence of AVP, the majority of AQP2 localizes to subapical vesicles [5], resulting in low water permeability of the principal cells. AVP binding to the basolateral AVP receptor initiates a signaling cascade leading to increased levels of cAMP and subsequent activation of Protein Kinase A (PKA). PKA phosphorylates AQP2 on S256 located in the cytoplasmic C-terminal tail [10] (Figure 1), which triggers plasma membrane accumulation of AQP2 [5,10]. This facilitates increased water transport and thus, urine concentration. Upon AVP removal, AQP2 localizes to intracellular vesicles resulting in decreased water uptake and diluted urine [5]. Long-term stimulation with AVP or thirst increases both AQP2 expression and apical targeting [11], while excessive water intake [11] and low levels of circulating vasopressin [12], decreases AQP2 expression and apical targeting.

Dysregulation of AQP2 as well as genetic mutations in AQP2 [23] and the AVP receptor [24,25,26,27], are key in many water balance disorders including nephrogenic diabetes insipidus (NDI), which is the inability to appropriately respond to circulating levels of AVP, acute kidney injury [28], chronic kidney injury [29], chronic heart failure [30], and many others.

It is thought that AQP2 constitutively cycles between intracellular vesicles and the plasma membrane. AQP2-S256A and AQP2-S256D mimics constitutively non-phosphorylated and phosphorylated AQP2, respectively. AQP2-S256A is predominantly localized to intracellular vesicles [31], whereas the constitutively phosphorylated form—AQP2-S256D—localizes to the plasma membrane [32]. AQP2-S256D is endocytosed upon inhibition of PKA as well as dopamine and Prostaglandin E2 (PGE2) stimulation, despite prior forskolin treatment to increase cAMP [20]. This indicates that S256 phosphorylation is not sufficient for plasma membrane localization. When endocytosis of AQP2-S256A was inhibited by expressing dominant negative dynamin 2, AQP2-S256A accumulated in the plasma membrane [33]. This indicates that AQP2-S256A is inserted into the plasma membrane but rapidly endocytosed. Moreover, it has been shown that internalization of AQP2-S256D is decreased compared to wild type AQP2 (AQP2-WT) [22,34]. Thus phosphorylation of AQP2 on S256 (p-AQP2-S256) seems to be essential for AQP2 plasma membrane retention.

In the renal collecting duct, the majority of p-AQP2-S256 localizes to the apical plasma membrane, but p-AQP2-S256 is also found in intracellular vesicles [35] indicating that there may be a certain threshold of p-AQP2-S256 phosphorylation needed to maintain AQP2 in the plasma membrane. Besides S256, S269 phosphorylation has also been shown to be involved in plasma membrane accumulation of AQP2, and both phosphomimics of S256 and S269 separately increased AQP2 protein stability, however, S256 phosphorylation is the dominant signal for AQP2 plasma membrane localization [36,37].

How a protein behaves in the plasma membrane depends on modifications, folding, protein-protein, protein-extracellular matrix (ECM)- and protein-lipid-interactions (for review please see [38,39]). Thus, diffusion of a protein in the plasma membrane can change when a protein is modified and potentially also change when a protein is destined for endocytosis versus residing in the plasma membrane. We still do not know the p-AQP2-S256 stoichiometry of plasma membrane localized AQP2 in epithelial cells. We hypothesized that AQP2 destined for endocytosis would diffuse at one particular speed, whereas AQP2 destined for plasma membrane localization would diffuse at a different speed. Using recently developed k-space Image Correlation Spectroscopy (kICS) [40,41,42,43] to measure diffusion coefficients of enhanced green fluorescent protein (EGFP)-tagged proteins, we aimed to measure diffusion of plasma membrane localized AQP2 at different degrees of AQP2-S256 phosphorylation using expression of different combinations of the phosphomutants AQP2-S256A and AQP2-S256D ranging from non-phosphorylated to complete phosphorylation of AQP2-S256. Our measurements show that diffusion of AQP2 changes with the number of phosphorylated monomers in a tetramer. When the tetramer only contains S256D phosphomimics, the tetramers diffuse significantly faster than non-phosphorylated S256A tetramers. When the tetramers contain an average of two to three phosphomimics, they diffuse like AQP2-S256D, whereas tetramers with only one phosphomimic diffuse like AQP2-S256A tetramers. Since we know that phosphorylation state determines plasma membrane retention and endocytosis rate, this difference in diffusion rate may reflect behavior of AQP2 tetramers destined for either plasma membrane retention or endocytosis.

## 2. Results and Discussion

### 2.1. Establishment of a Cell Culture System for Measurements of Average Diffusion Coefficients of Aquaporin-2

**Figure 2:**
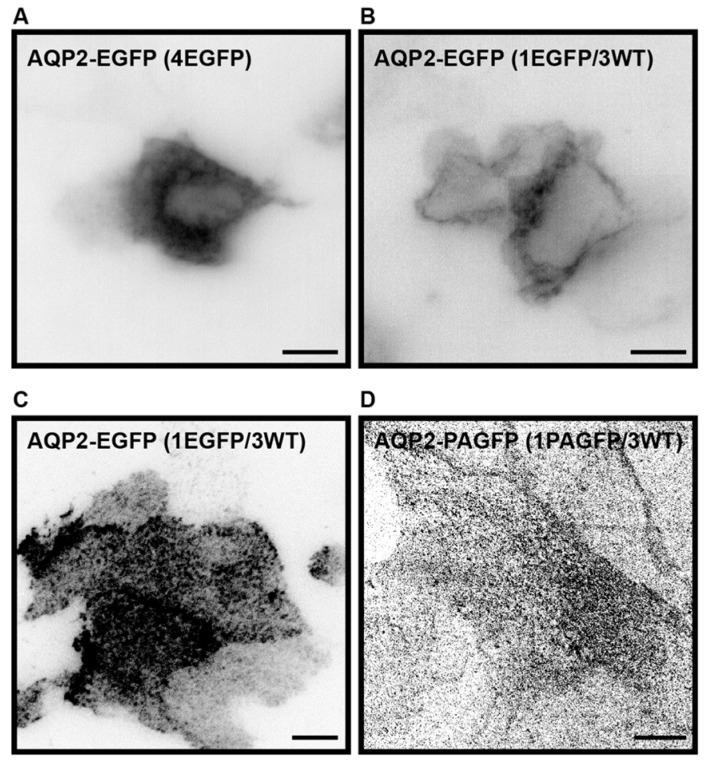
AQP2 plasma membrane localization is rescued by co-transfection of AQP2-EGFP (enhanced green fluorescent protein) and untagged AQP2. (**A**,**B**) Representative fluorescence images of average projections of a Z-stack of MDCK cells transiently transfected with AQP2-EGFP (**A**) and a mixture of AQP2-EGFP and untagged AQP2 in the ratio 1:3 (**B**), hereafter named AQP2-wild type (AQP2-WT). Fluorescence is shown in inverted contrast; (**C**) representative fluorescence image captured in Total Internal Reflection Fluorescence (TIRF) mode of MDCK cells transiently transfected with a mixture of AQP2-EGFP and AQP2-WT in the ratio 1:3. Fluorescence is shown in inverted contrast; (**D**) Photoactivatable Localization Microscopy (PALM) image rendering of MDCK cells transiently transfected with AQP2-PAGFP (photoactivatable green fluorescent protein) and untagged AQP2 in the ratio 1:3. Peaks were found in 7000 frames using a peak selector. Scale bars are 10 μm.

Previously, AQP2-EGFP has been stably expressed in LLC-PK1 cells, where it localized correctly to the plasma membrane and intracellular vesicles [44]. We obtained the same construct used to generate the stable LLC-PK1 AQP2-EGFP cell line, but found that when expressed in MDCK cells, AQP2-EGFP had a tendency to aggregate (Figure 2A). To overcome aggregation, we co-expressed AQP2-EGFP and non-tagged AQP2-WT in a plasmid ratio of one AQP2-EGFP to three non-tagged AQP2-WT. With this ratio, the number of EGFP-tagged AQP2 monomers per AQP2 tetramer is reduced to an average of one. Co-expression of untagged AQP2-WT with AQP2-EGFP is named AQP2-WT throughout the manuscript. Co-expression of tagged and un-tagged AQP2 has previously been shown to result in the generation of heterotetramers, which traffic normally to the plasma membrane [45]. We also found that the co-expression resulted in localization to the plasma membrane and intracellular vesicles (Figure 2B), as previously observed in the stable AQP2-EGFP expressing LLC-PK1 cell line [44]. AQP2-WT localized to the basal plasma membrane of subconfluent cells and could be imaged using Total Internal Reflection Fluorescence (TIRF) (Figure 2C) as well as Photoactivatable Localization Microscopy (PALM), [46,47] EGFP was replaced by photoactivatable GFP (PAGFP) for PALM, (Figure 2D).

MDCK cells are frequently used as a model system to study polarized epithelial cells in culture. They were derived from canine kidney, are homogeneous and when grown on filters, they polarize, forming apical and basolateral membranes with correct trafficking of apical and basolateral plasma membrane proteins (for review [48]), including AQPs [49]. To perform k-space Image Correlation Spectroscopy (kICS) analysis and measure AQP2 diffusion in the plasma membrane, cells were grown subconfluently on glass, which allows imaging of the basal membrane and selection of large crops for analysis [43]. Such imaging is not possible to perform on filters.

In some studies of AQP2 shuttling, the cAMP reducing agent—indomethacine—is added to the medium, to reduce plasma membrane levels of AQP2 prior to e.g., forskolin stimulation [20]. However, to mimic the in vivo conditions, where some AQP2 is found in the plasma membrane we chose not to include indomethacine.

Thus, we successfully set up a cell culture system, where AQP2 tetramers can be imaged in epithelial cells.

### 2.2. Plasma Membrane Diffusion of AQP2 Changes with the Degree of S256 Phosphorylation

It has been shown that AQP2-S256(4D) localizes to the plasma membrane [20,32] and is prone to plasma membrane retention as well as protein stability [22], whereas AQP2-S256(4A) is prone to rapid endocytosis [33]. Moreover, in *Xenopus* oocytes, three monomers in the tetramer needed to be phosphorylated at position S256 to target AQP2 from intracellular vesicles to the plasma membrane [45]. However, the stoichiometry of AQP2-S256 phosphorylation in the plasma membrane in epithelial cells is unknown. We thus wanted to measure diffusion coefficients in respect to the degree of AQP2 phosphorylation at position S256 to potentially reveal differences correlating to the S256 phosphorylation state.

**Figure 3:**
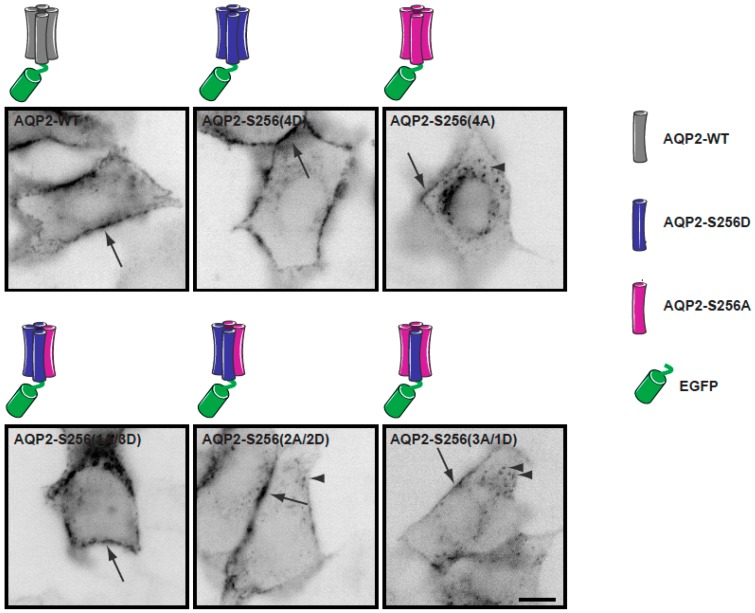
Subcellular localization of the different homo- and heterotetramers mimicking different degrees of Aquaporin-2-S256 (AQP2-S256) phosphorylation. Representative fluorescence images of MDCK cells transiently transfected with the indicated combinations of AQP2 plasmids. Images are shown in inverted contrast. Arrows and arrow heads point to AQP2 localized in the plasma membrane and intracellular vesicles, respectively. Scale bar is 10 μm. Schematics depict the different homo- and heterotetramers used in this study. The monomers corresponding to AQP2-WT are colored in grey and the monomers of AQP2-S256D and AQP2-S256A are colored in blue and magenta, respectively. In each tetramer, one of the monomers is tagged with an N-terminal EGFP, (green).

To measure diffusion coefficients, cells transiently transfected with the different combinations of AQP2, AQP2-S256A and AQP2-S256D (indicated in Figure 3) were seeded on collagen coated coverslips and allowed to attach and spread for 48 h.

When imaging the different combinations using widefield microscopy, it was found that AQP2 localization to the plasma membrane and intracellular vesicles correlated with the phosphorylation level of S256 (Figure 3) as expected. Indeed, few intracellular vesicles were observed for AQP2-S256(4D) (Figure 3). On the other hand, AQP2-S256(4A) (Figure 3), displayed a higher localization to intracellular vesicles as has been shown before [31,45].

**Figure 4:**
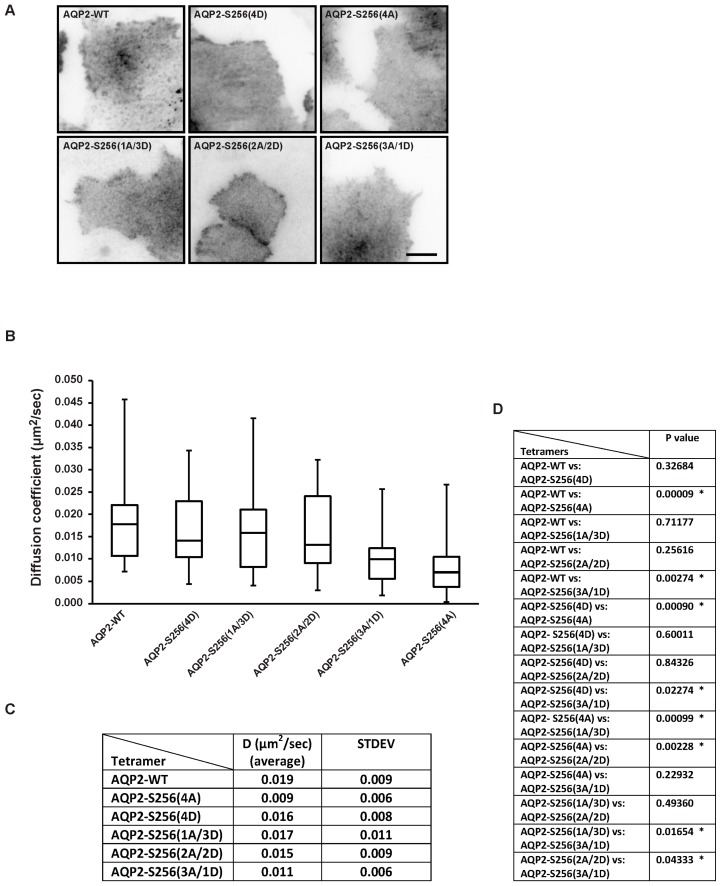
AQP2 diffusion coefficients change with respect to the degree of S256 phosphorylation. (**A**) Representative fluorescence images captured in Total Internal Reflection Fluorescence (TIRF) mode of MDCK cells transiently transfected with the indicated constructs. Fluorescence is shown in inverted contrast. Scale bar is 10 μm; (**B**) k-space Image Correlation Spectroscopy (kICS) analysis of time-lapse image sequences of MDCK cells transiently transfected with untagged AQP2, AQP2-S256(4D), AQP2-S256(4A), AQP2-S256(1A/3D), AQP2-S256(2A/2D), and AQP2-S256(3A/1D). Diffusion coefficients are shown in a Box-and-whiskers plot; (**C**) table showing average diffusion coefficients and standard deviations (STDEV) for each combination of transfection combination; (**D**) table showing *p*-values. * indicates *p* < 0.05.

In contrast to the differences in distribution between the plasma membrane and intracellular vesicles observed by wide field microscopy (Figure 3), there was no detectable difference in the localization when the basal membrane was imaged in TIRF mode (Figure 4A). The AQP2 detected in the TIRF zone corresponds to plasma membrane embedded AQP2 and some AQP2 in intracellular vesicles just above the basal membrane may also be visualized. To measure diffusion coefficients, fast time-lapse sequences were acquired in TIRF and image files were loaded into MATLAB (MathWorks, Natick, MA, USA) for analysis using the kICS code. Crops used for kICS analysis were chosen in the flat region of the plasma membrane and were selected so that no moving vesicles, holes, or drift could be observed [40,43].

kICS analysis revealed that the average diffusion coefficients for AQP2-WT and AQP2-S256(4D) were not significantly different, however, both were significantly different from the diffusion coefficient of AQP2-S256(4A), which diffused significantly slower than AQP2-WT and AQP2-S256(4D) (Figure 4B–D: (B) Box-and-whiskers plot; (C) average diffusion coefficients and standard deviations listed in table format; (D) *p* values, *p* < 0.05 is marked by * and considered significant).

AQP2-S256(4D) has been shown to have longer plasma membrane lifetime and stability [20,22,32] compared to AQP2-S256(4A), which is destined for rapid endocytosis [21,31,33].

To measure at which AQP2-S256 phosphorylation state this change in diffusion occurs, we measured diffusion coefficients of AQP2 in cells transfected with different ratios of AQP2-S256A and AQP2-S256D. Transfections using different ratios of plasmids encoding AQP2 phosphomimics to obtain tetramers of different combinations of phosphorylation states have previously been confirmed by immunoblotting [45].

Our data showed (Figure 4B–D) that AQP2-S256(2A/2D) and AQP2-S256(1A/3D) diffused at rates similar to those of AQP2-WT and AQP2-S256(4D), but significantly different than AQP2-S256(4A). In contrast, AQP2-S256(3A/1D) diffused at a rate similar to AQP2-S256(4A), but significantly slower than AQP2-WT and AQP2-S256(4D)/(1A/3D)/(2A/2D). This indicates that when a minimum of two AQP2 monomers within the tetramer are phosphorylated at S256, the tetramer diffuses as AQP2-S256(4D). Future studies will clarify if changes in diffusion of AQP2 tetramers are involved in plasma membrane retention and decreased endocytosis.

A previous study using fluorescence recovery after photobleaching (FRAP) analysis of EGFP-AQP2 expressed in subconfluent LLC-PK1 cells and MDCK cells, found that diffusion of EGFP-AQP2-WT was equal to that of EGFP-AQP2-S256A [50]. Compared to the capability of the kICS analysis presented in this study, the used FRAP system and analysis are slow, and may not have captured fast moving fractions of AQP2. Moreover, the FRAP analysis was performed on the lateral plasma membrane, where diffusion may differ compared to the basal membrane.

## 3. Materials and Methods

### 3.1. Plasmids, Bacterial Growth Conditions, and Cell Culture

The plasmids encoding Rat AQP2-c-myc-EGFP (AQP2*-pEGFP-) [44] and AQP2-c-myc (pcDNAI-AQP2*) [51] were kindly provided by Dr. Dennis Brown, Harvard Medical School, Boston, MA, USA. After sequencing, we identified a I54T (ATC to ACC) point mutation that was corrected by site directed mutagenesis to work with the canonical Rat AQP2 sequence. The AQP2-WT-PAGFP-construct was generated by Eurofins Genomics (Eurofins Genomics, Ebersberg, Germany). Standard molecular biology methods were used to generate the other plasmid constructs. The different plasmids used are listed in the supplemental material (Table S1). The PCR primers used in the cloning strategies are listed in Table S2. The sequences of all the constructs were systematically verified (GATC Biotech, Constance, Germany).

*Escherichia coli* strain NM522 (New Englands Biolabs, Ipswich, MA, USA) was used for cloning and plasmid amplification. Bacteria were grown in Luria-Bertani broth (LB) or on Luria agar plates at 37 °C. Antibiotics were added to the medium for selection according to the resistance markers carried by the plasmid. The following concentrations were used: kanamycin, 50 μg/mL and Ampicillin, 100 μg/mL.

MDCK GII cells [52,53] were grown in dulbeccos modified eagles medium (DMEM) with low glucose (1 g/L, Gibco, Copenhagen, Denmark) with 10% fetal bovine serum (FBS, Gibco, Copenhagen, Denmark) and 0.5 U/mL penicillin (Sigma, Copenhagen, Denmark), 0.5 g/mL streptomycin (Gibco, Copenhagen, Denmark), and 1 mg/mL kanamycin (Gibco, Copenhagen, Denmark) at 37 °C and 5% CO_2_.

Transfections were performed in the ratio of one GFP-tagged AQP2 to three non-tagged AQP2 using Lipofectamine 2000 (Invitrogen, ThermoFisher SCIENTIFIC, Hvidovre, Denmark). Different combinations of AQP2-S256A and AQP2-S256D were used to mimic different phosphorylation states of the four monomers (see Figure 3). The DNA constructs were transiently expressed in MDCK GII cells grown on collagen coated coverslips with no apparent change in phenotype.

### 3.2. Microscopy

For live-cell TIRF microscopy, cells were transfected and seeded on rat-tail collagen coated coverslips two days before imaging. For imaging, media was changed to phenol red-free DMEM (Sigma-Aldrich, Copenhagen, Denmark) with 10% FBS, 1% PSK, 4 mM l-glutamine and 25 mM HEPES. Imaging was performed using a Nikon T*i* Eclipse automated TIRF microscope (Ramcon A/S, Birkerød, Denmark). For detection, an Andor iXon EMCCD (Andor Technology Ltd., Belfast, UK) was used. Imaging was performed using a 488 nm laser line for excitation using Total Internal Reflection Fluorescence (TIRF) mode. The temperature was 37 °C. A 100× Apo oil TIRF objective (NA 1.49) was used. Stacks composed of 500 frames were acquired with 60 ms integration time at an average frame rate of 14.1 Hz. Experiments were repeated three times and cells from each experiment were included in the analysis. A minimum of seven cells from each day were analyzed per transfection combination, thus in total 21–25 crops per transfection combination were analyzed.

Images of fixed cells were acquired on a Nikon T*i* Eclipse inverted fluorescence microscope equipped with a pE-300 WHITE LED illumination unit using a Plan Apo objective (100×/1.45 NA) (Ramcon A/S, Birkerød, Denmark). Acquisition was done with an Andor Zyla 5M pixel camera (Andor Technology Ltd., Belfast, UK). For GFP a 469/35 excitation filter and a 525/39 emission filter was used.

### 3.3. Sample Preparation for Photoactivated Localization Microscopy (PALM) Imaging

Coverslips (#1.5) were cleaned in 2% Helmanex III (Fisher Scientific, Copenhagen, Denmark) for 3 h and then dipped in MilliQ water 10 times followed by sterilization in 99% Ethanol for 20 min. Cleaned coverslips were coated with rat-tail collagen. MDCK cells were transfected in suspension with AQP2-WT and AQP2-PAGFP (3:1) 48 h before imaging. On the day of imaging, cells were washed at room temperature in phosphate-buffered saline (PBS) followed by 30 min fixation in 4% paraformaldehyde (PFA) (Electron Microscopy Sciences, Hatfield, PA, USA) and 0.1% glutaraldehyde (Electron Microscopy Sciences) in PBS. Fluorescence from PFA was quenched with 50 mM glycine (Sigma) in PBS twice for 5 min. Sonicated 0.1 μm tetraspecs (Invitrogen) diluted 1:2000 in PBS were added to the sample and left for 5 min, imaging was performed in PBS.

### 3.4. Photoactivatable Localization Microscopy (PALM) Imaging

Fixed PALM was performed on a Nikon T*i* Eclipse, automated Total Internal Reflection Fluorescence setup equipped with an Andor EMCCD camera and 405 and 488 nm lasers. 8000 frames were acquired with the 488 nm laser with a continuously low level of photoactivation of PAGFP from the 405 nm laser. Exposure time was 100 ms. Image rendering of the last 7000 frames were created using Peak Selector [54], which is an algorithm written in IDL (Excelis Visual Information Solutions). Peaks identified in each frame were fitted using a Gaussian point spread function. Images were drift corrected and rendered in Peak Selector.

### 3.5. Measurement of Diffusion Coefficients by k-Space Image Correlation Spectroscopy (kICS) Analysis

Image stacks were imported into ImageJ [55] and crops with no moving cell organelles and no moving membrane were selected. The crops were analyzed using the kICS code in MATLAB [40]. The same settings were used for all crops; the maximum number of time lags (τ) was set to 5 and the maximum k^2^ value was set to 40. The diffusion coefficients were imported into Excel, and a Box-and-whiskers plot was generated. Also, average diffusion coefficients and standard deviations were calculated. Experiments were repeated three times and a total of crops from 21 to 25 cells per transfection combination were analyzed by kICS.

### 3.6. Statistics

Comparisons between groups were made by ANOVA followed by unpaired *t*-test. *p*-values < 0.05 were considered significant.

## 4. Conclusions

We successfully set up a cell system allowing us to express and image AQP2 and phosphorylation variants thereof. AQP2 aggregation due to EGFP was rescued by co-transfection of AQP2-EGFP and untagged AQP2. Using previously published methods of high-speed imaging combined with kICS analysis, diffusion coefficients of AQP2-WT and phosphorylation variants were extracted in order to investigate the influence of the number of phosphorylated monomers. When AQP2 tetramers contained an average of two to four S256 phosphomimics, the tetramers diffused at a higher speed than when the tetramers contained only one or no phosphomimics. Future studies will clarify if this difference in diffusion rate may reflect behavior of AQP2 tetramers destined for either plasma membrane retention or endocytosis in relation to the S256 phosphorylation state.

## Supplementary Materials

Supplementary materials can be found at www.mdpi.com/1422-0067/17/11/1804/s1.
