# Supplementary material for: AQP2 Plasma Membrane Diffusion Is Altered by the Degree of AQP2-S256 Phosphorylation
Source: Int J Mol Sci. 2016 Oct 28;17(11):1804. doi: 10.3390/ijms17111804 (PMC5133805; doi:10.3390/ijms17111804)
Supplement: Supplementary file 1 [file ijms-17-01804-s001.pdf]

Supplementary Materials: AQP2 Plasma Membrane Diffusion Is Altered by the Degree of AQP2-S256 Phosphorylation

Eva C. Arnspang, Frédéric H. Login, Jennifer S. Koffman, Prabuddha Sengupta and Lene N. Nejsum

Table S1. Plasmids used in this study.

Plasmids	Description [a]	Reference
pEGFP-AQP2*	$AQP2^{I54T}$ with a C-terminal c-Myc tag in pEGFP-C1. EGFP fused to the N-terminus of $AQP2^{I54T}$ that has a C-terminal c-Myc tag, Km^r.	[37]
pcDNAI-AQP2*	$AQP2^{I54T}$ with a C-terminal c-Myc tag in pcDNAI/Neo. $AQP2^{I54T}$ with a C-terminal c-Myc tag, Km^r.	[38]
pEGFP-$AQP2^{wt}$	pEGFP-AQP2* without the I54T mutation.	This study
pcDNAI-$AQP2^{wt}$	pcDNAI-AQP2* without the I54T mutation.	This study
pEGFP-$AQP2^{S256D}$	pEGFP-$AQP2^{wt}$ with S256D point mutation. This construct is annotated AQP2-S256D-EGFP in the text.	This study
pEGFP-$AQP2^{S256A}$	pEGFP-$AQP2^{wt}$ with S256A point mutation. This construct is annotated AQP2-S256A-EGFP in the text.	This study
pcDNA3.1/zeo(+)	Mammalian expression vector, CMV promoter, Amp^r.	Invitrogen
pcDNA-$AQP2^{wt}$	$AQP2^{wt}$ with a C-terminal c-Myc tag subcloned from pcDNAI-$AQP2^{wt}$ into pcDNA3.1/zeo(+) using *Xho*I and *Xba*I. This construct is annotated AQP2 WT in the text.	This study
pcDNA-$AQP2^{S256D}$	pcDNA-$AQP2^{wt}$ with S256D point mutation. This construct is annotated AQP2-S256D in the text.	This study
pcDNA-$AQP2^{S256A}$	pcDNA-$AQP2^{wt}$ with S256A point mutation. This construct is annotated AQP2-S256A in the text.	This study
pPAGFP-C1	Mammalian expression vector for N-terminal fusion with photoactivatable GFP (PAGFP) , Km^r.	[46]
pPAGFP-$APQ2^{wt}$	$AQP2^{wt}$ with a C-terminal c-Myc tag in pPAGFP-C1. This construct is annotated AQP2 WT-PAGFP in the text.	This study

[a] Km^r, kanamycin resistance; Amp^r, Ampicillin resistance.

Table S2. Primers used in this study.

Primer Name	Primer Sequence (5′–3′)	Description
F-CMV	cgcaaatgggcggtaggcgtg	For amplification with pcDNA-$AQP2^{wt}$ as template
R-BGH	actagaaggcacagtcgaggctg	
F-EGFP-C	catggtcctgctggagttcgtg	For amplification with pEGFP-AQP2 as template
R-pBABE	accctaactgacacacattcc	
R-AQP2_S256D	gaggagagtggagctccacgtcctgccgccgccgcact	To introduce S256D point mutation
F- AQP2_S256D	agtgcggcggcggcaggacgtggagctccactctcctcag	
R-AQP2_S256A	ctgaggagagtggagctccactgcctgccgccgccgcact	To introduce S256A point mutation
F-AQP2_S256A	agtgcggcggcggcaggcagtggagctccactctcctcag	
